# Relationship between elimination disorders and internalizing‐externalizing problems in children: A systematic review and meta‐analysis

**DOI:** 10.1002/jcv2.12185

**Published:** 2023-07-27

**Authors:** Claudia Aymerich, Borja Pedruzo, Malein Pacho, Jon Herrero, María Laborda, Marta Bordenave, Gonzalo Salazar de Pablo, Eva Sesma, Aranzazu Fernández‐Rivas, Ana Catalan, Miguel Ángel González‐Torres

**Affiliations:** ^1^ Psychiatry Department Basurto University Hospital Bilbao Spain; ^2^ Psychiatry Department. Biocruces Bizkaia Health Research Institute Bilbao Spain; ^3^ Centro de Investigación en Red de Salud Mental (CIBERSAM) Madrid Spain; ^4^ Department of Child and Adolescent Psychiatry Institute of Psychiatry, Psychology & Neuroscience King's College London London UK; ^5^ Department of Psychosis Studies Early Psychosis: Interventions and Clinical‐detection (EPIC) Lab Institute of Psychiatry, Psychology and Neuroscience King's College London London UK; ^6^ Child and Adolescent Mental Health Services (CAMHS) South London and Maudsley NHS Foundation Trust London UK; ^7^ Neuroscience Department University of the Basque Country (UPV/EHU) Leioa Spain; ^8^ Department of Psychosis Studies Institute of Psychiatry, Psychology and Neuroscience King's College London London UK

**Keywords:** comorbidities, elimination disorder, encopresis, enuresis, externalizing, internalizing, self‐concept

## Abstract

**Background:**

Elimination disorders are highly prevalent in childhood and often associated with clinically relevant comorbid psychological disorders. The aim of this study is to determine if, and to what extent, children with elimination disorders show higher internalizing and externalizing problems than their healthy peers.

**Methods:**

A multistep literature search was performed from database inception until May 1st, 2022. PRISMA/MOOSE‐compliant systematic review (PROSPERO: CRD42022303555) were used to identify studies reporting on internalizing and/or externalizing symptoms in children with an elimination disorder and a healthy control (HC) group. First, a systematic review was provided. Second, where data allowed for it, a quantitative meta‐analysis with random effects model was conducted to analyze the differences between the elimination disorder and the HC groups for internalizing and externalizing symptoms. Effect size was standardized mean difference. Meta‐regression analyses were conducted to examine the effect of sex, age, and study quality. Funnel plots were used to detect a publication bias. Where found, the trim and fill method was used to correct it.

**Results:**

36 articles were included, 32 of them reporting on enuresis (*n* = 3244; mean age = 9.4; SD = 3.4; 43.84% female) and 7 of them on encopresis (*n* = 214; mean age = 8.6; SD = 2.3; 36.24% female). Children with an elimination disorder presented significantly lower self‐concept (ES:0.42; 95%CI [0.08; 9.76]; *p* = 0.017) and higher symptom scores for thought problems (ES:−0.26; 95%CI: −0.43;−0.09]; *p* = 0.003), externalizing symptoms (ES: −0.20; 95%CI [−0.37;−0.03]; *p* = 0.020), attention problems (ES:−0.37; 95%CI [−0.51;−0.22]; *p* = 0.0001), aggressive behavior (ES:−0.33; 95%CI [−0.62;−0.04]; *p* = 0.025) and social problems (ES:−0.39; 95%CI [−0.58;−0.21]; *p* = 0.0001). Significant publication biases were found across several of the studied domains. No significant effect of sex, age or quality of the study score was found.

**Conclusions:**

Children with an elimination disorder may have significant internalizing and externalizing problems, as well as impaired self‐concept. It is recommendable to screen for them in children with enuresis or encopresis and provide appropriate interventions.

## Graphical abstract

This is the first systematic review and meta‐analysis to assess the relationship between elimination disorders and internalizing and externalizing symptoms. Enuresis is associated to lower self‐concept and higher symptom scores for thought problems, externalizing symptoms, attention problems, aggressive behavior and social problems.

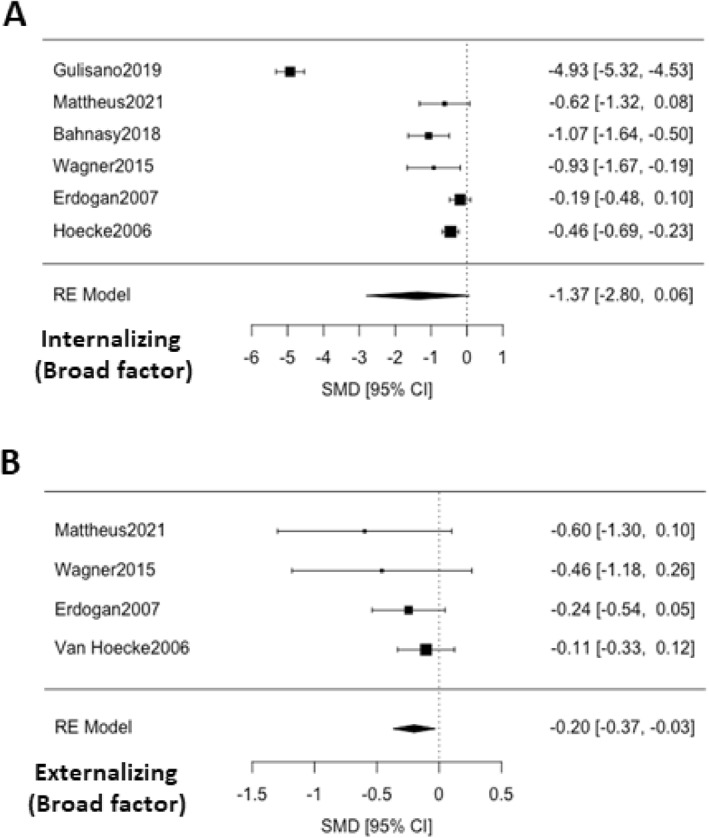

**Key Points**

- What's known: Elimination disorders are highly prevalent among children, and often associated with clinically relevant psychological comorbid disorders.
- What's new: This is the first systematic review to assess the relationship between elimination disorders and internalizing and externalizing symptoms. Such disorders are associated to lower self‐concept and higher symptom scores for thought problems, externalizing symptoms, attention problems, aggressive behavior and social problems. Most available studies assessing this topic are at high risk for bias.
- What's relevant: Emotional and behavioral problems should be considered when evaluating children with enuresis or encopresis. Further research is needed to clarify the relationship between internalizing and externalizing symptoms and elimination disorders and the factors moderating it.

## INTRODUCTION

### Background

Elimination disorders include enuresis, defined as wetting from 5 years, and encopresis, defined as soiling from 4 years onwards after organic causes are excluded (American Psychiatric Association, 2022). They constitute a very common group of disorders in childhood with similar rates worldwide; 1%–3% of 4‐year‐olds are affected by encopresis, while up to 10% of 7‐year‐olds suffer from nocturnal enuresis (Reese et al., 2013). Both conditions significantly decrease in prevalence over late childhood and adolescence, with a spontaneous rate of approximately 15% per year (Tai et al., 2016).

As interest in the physiopathology and etiology of elimination disorders has grown in the last decade, it has been described that psychological disorders are several times more frequent in children with enuresis or encopresis: the latest research indicates that 20%–30% of children with enuresis and 30%–50% with encopresis have clinically relevant comorbid disorders (vonGontard et al., 2011), which represents over a 4‐times increase compared with their healthy peers. The most common comorbid disorder in both encopresis and enuresis is ADHD (deSena Oliveira et al., 2021), followed closely by oppositional defiant disorder (Joinson et al., 2006). Other worrying findings include that patients and family members of patients with enuresis showed worse quality of life global scores when compared to healthy control children, similar to those found in individuals with chronic conditions such as asthma or epilepsy (Bachmann et al., 2009). Less is known, however, on the prevalence and severity of subclinical behavioral symptoms that cannot be technically classified as a disorder, such as sadness, embarrassment or aggressive behavior.

Internalizing and externalizing problems generally describe two broad groupings of behavioral, emotional, and social problems (Achenbach et al., 2016), conforming rather heterogeneous categories. Internalizing problems refer to inwardly focused negative behaviors, including anxiety, depression, and withdrawn conduct. On the other hand, externalizing problems describe outwardly focused negative behaviors such as aggression and disruptive conduct (Liu, 2004). Internalizing and externalizing problems during childhood predict later negative adolescent and adult psychological and physical health outcomes (Tien et al., 2019).

Although some previous systematic reviews examine the relationship and comorbidity between elimination disorders and specific psychiatric conditions (deSena Oliveira et al., 2021), there is no systematic review or meta‐analysis examining their co‐occurrence with internalizing and externalizing problems in children. We consider it to be a highly relevant topic both in terms of prevalence and the high psychological burden associated to these disorders.

### Objectives

In this review we systematically researched the literature in order to try to answer the following research questions:

1. To what extent do children with elimination disorders show higher internalizing and externalizing problems than controls (defined as age‐matched healthy peers)?
2. Is the group standardized difference between elimination disorders and controls in the internalizing and externalizing problems moderated by (a) the nature of the primary disorder, age or sex? or (b) methodological factors, including sample size, or publication bias?

## METHODS

This study protocol was registered on PROSPERO (registration number: CRD42022303555). The study was conducted in accordance with “Preferred Reporting Items for Systematic Reviews and Meta‐Analyses” (PRISMA) (Moher et al., 2009) (See Table S1) and “Meta‐analyses of Observational Studies in Epidemiology” (MOOSE) checklist (Stroup et al., 2000) (See Table S2), following “EQUATOR Reporting Guidelines” (Altman et al., 2008).

### Search strategy and selection criteria

A systematic literature search was carried out by two independent researchers (C.A. and B.P.). Web of Science database (Clarivate Analytics) was searched, incorporating the Web of Science Core Collection, the BIOSIS Citation Index, the KCI‐Korean Journal Database, MEDLINE®, the Russian Science Citation Index, and the SciELO Citation Index as well as Cochrane Central Register of Reviews, and Ovid/PsycINFO databases, from inception until May 1^st^, 2022.

The following keywords were used: “encopresis” OR “enuresis” OR “elimination disorder*” AND “anxiety” OR “depression” OR “agressi*” OR “rule‐break*” OR “conduct” OR “opposition*” OR “self‐concept” OR “self‐esteem” OR “internalizing” OR “externalizing”.

Articles identified were first screened as abstracts, and after excluding those that did not meet the inclusion criteria, the full texts of the remaining articles were assessed for eligibility and inclusion. In case of disagreement a senior researcher (A.C.) made the final decision on the inclusion of the article.

Inclusion criteria for the systematic review and meta‐analysis were: (a) individual studies with original data, (b) focusing on participants up to 18 years of age with a clinical diagnosis of an elimination disorder (including enuresis, encopresis, or a combination of both) provided by specialists, (c) reporting quantitative data on internalizing and/or externalizing problems (including anxiety, depression, or a combination of both, aggressive behaviors, rule‐breaking behaviors, conduct problems, oppositional defiance, or their combination), (d) using validated, structured evaluation scales evaluating internalizing or externalizing problems as continuous variables through standard self‐report or parents‐report tools, (e) reporting cross‐sectional data or longitudinal data on internalizing and externalizing problems in children with an elimination disorder and a comparison group of age‐matched healthy controls children (healthy controls) on the outcomes of interest, and (f) written in English or Spanish. Exclusion criteria were (a) reviews, clinical cases, study protocols or qualitative studies, conferential proceedings, letters, and commentaries, (b) reporting on patients with cognitive impairment, and (c) written in languages other than English or Spanish.

### Data extraction

Three researchers (M.P., M.L., and J.H.) independently extracted data from all the included studies. The three databases were then cross‐checked, and discrepancies were resolved through consensus under the supervision of a senior researcher (A.C.). A summary of selected variables included: first author and year of publication, country and city, sample size, mean age (mean ± standard deviation [SD]), primary disorder (enuresis, encopresis, or a combination of both), specifiers of the elimination disorder (e.g., diurnal, nocturnal), sex (% female), pharmacological treatment (as a dichotomic variable, when more than 30% of the clinical group were receiving pharmacological treatment for the elimination disorder), outcomes of interest (including the category, the nature of the internalizing or externalizing symptom, and the rating tool used to assess them), quality assessment (see below), and key findings.

When multiple data points were available, the point in which the elimination disorder was diagnosed was coded. For longitudinal studies, data for the first time point were extracted.

Studies were examined for potential sample overlap. When studies had a high sample overlap (determined by looking at the inclusion dates, type of population and country in which the study was carried out), the study with the largest sample was selected.

### Risk of bias (quality) assessment

Risk of bias was assessed using Newcastle‐Ottawa Scale for cross‐sectional and cohort studies (Wells et al., 2012) (See Table S3). No article was excluded from the systematic review or meta‐analyses regardless of their NOS score.

### Certainty of evidence assessment

Certainty of evidence of the included studies was assessed using the GRADE approach: Grading of Recommendations Assessment, Development and Evaluation (Higgins et al., 2022).

### Strategy for data synthesis and statistics

First, we provided a systematic synthesis (Table 1 and Table 2) of the findings from the included studies.

**TABLE 1:** Articles included in the systematic review for the enuresis sample.

Author year	Country	N EN	N HC	Age mean (SD)	% Female	NT/CB	Medicaltreatment	Domain (scale)	EN mean (SD)/cutoff %	HC mean (SD)/cutoff %	NOS
Gulisano et al. (2016)	Italy	200	200	11.7 (3.3)	36.7	NT	No	Internalizing (CBCL) Externalizing (CBCL) Withdrawn behavior (CBCL) Somatic complaint (CBCL) Anxious/Depressed (CBCL) Social problems (CBCL) Thought problems (CBCL) Attention problems (CBCL) Delinquent behavior (CBCL) Aggressive behavior (CBCL) Depression (CDI) Anxiety (MASC)	50.5 (8.9) 6.7 (3.8) 15.2 (2.3) 14.8 (4.6) 20.0 (3.3) 2.1 (0.9) 1.8 (0.7) 18.0 (1.1) 3.5 (2.8) 2.9 (1.1) 10.3 (6.1) 52.9 (8.3)	13.6 (5.7) 6.5 (2.9) 3.7 (2.2) 4.3 (5.1) 5.6 (2.8) 1.9 (1.2) 1.7 (0.9) 4.0 (1.6) 4.1 (1.9) 3.0 (1.2) 6.8 (5.1) 40.9 (12.7)	8
van Hoecke et al. (2003)	Belgium	154	153	8.5 (1.8)	38.1	CB	No	Internalizing (CBCL) Externalizing (CBCL) Withdrawn behavior (CBCL) Somatic complaint (CBCL) Anxious/Depressed (CBCL) Social problems (CBCL) Thought problems (CBCL) Attention problems (CBCL) Delinquent behavior (CBCL) Aggressive behavior (CBCL) Attention problems (DBDRS) Hyperactive problems (DBDRS)	56.9 (11.1) 52.5 (11.2) 2.4 (2.5) 1.4 (2.0) 5.0 (4.6) 2.4 (2.5) 0.8 (1.3) 5.2 (3.9) 1.6 (2.2) 8.0 (6.6) 7.4 (5.9) 6.9 (6.3)	53.4 (10.5) 49.2 (11.3) 1.5 (1.9) 1.3 (1.9) 3.4 (3.7) 1.8 (2.3) 0.3 (0.7) 3.4 (3.3) 1.0 (1.3) 5.6 (5.0) 5.7 (5.4) 4.7 (5.4)	6
Niemczyk et al. (2014)	Germany	134	1466	5.7 (0.4)	50.2	CB	N.a.	Externalizing (DYSPIS)	13.4%	9.5%	6
De Bruyne et al. (2009)	Belgium	78	110	8.8 (2.0)	45.2	CB	No	Internalizing (CBCL) Externalizing (CBCL) Withdrawn behavior (CBCL) Somatic complaint (CBCL) Anxious/Depressed (CBCL) Social problems (CBCL) Thought problems (CBCL) Attention problems (CBCL) Delinquent behavior (CBCL) Aggressive behavior (CBCL) Attention problems (DBDRS) Hyperactive problems (DBDRS)	7.9 (8.1) 9.3 (9.7) 2.5 (2.6) 1.3 (2.1) 4.5 (5.0) 2.2 (2.7) 0.8 (1.9) 4.9 (4.7) 1.2 (1.8) 8.0 (8.2) 6.8 (7.1) 5.1 (6.2)	6.5 (7.4) 6.0 (7.8) 1.7 (2.3) 1.5 (2.0) 3.5 (4.5) 1.5 (2.2) 0.5 (1.1) 3.1 (3.3) 1.1 (2.1) 4.9 (6.0) 3.6 (4.4) 3.0 (3.6)	6
Azimi et al. (2019)	Iran	100	100	7.3 (1.8)	39.5	NT	No	Anxiety (SCARED)	22.0%	6.0%	6
Karaca Ünlü et al. (2020)	Turkey	52	44	9.7 (N.a.)	N.a.	NT	No	Anxiety (SCARED)	32.5 (10.7)	26.7 (10.6)	9
Mattheus et al. (2021)	Germany	10	42	5.6 (0.8)	40.4	CB	No	Internalizing (CBCL) Externalizing (CBCL) Depression (PFC)	55.4 (10.7) 51.9 (15.4) 1.3 (2.1)	44.8 (10.5) 44.3 (11.7) 0.7 (1.3)	7
Coppola et al. (2011)	Italy	22	22	9.8 (3.0)	37.37	CB	Yes	Self‐esteem (MSCS) Behavioral problems (SDQ)	89.3 (8.7) 14.2 (5.8)	110.1 (12.0) 6.2 (4.1)	5
Theunis et al. (2002)	Belgium	50	77	9.5 (N.a.)	N.a.	N.a.	Yes	Self‐esteem (SPPC)	18.3 (3.3)	20.1 (3.3)	4
Ertan et al. (2009)	Turkey	44	27	10.4 (3.0)	N.a.	NT	No	Sleep (PSQI)	3.6 (2.1)	3.4 (1.7)	6
Robinson et al. (2003)	United Kingdom	25	25	9.9 (2.0)	52.0	NT	No	Self‐esteem (CSEI)	62.4 (17.7)	67.8 (17.9)	6
Yaradilmiş et al. (2020)	Turkey	120	109	9.1 (2.2)	27	NT	No	Depression (CDI)	9.5 (6.0)	7.8 (5.6)	5
Gozmen et al. (2008)	Turkey	19	32	9.5 (1.5)	43.1	NT	Yes	Sleep (PSQI)	5.5 (2.7)	2.5 (1.0)	7
Bahnasy et al. (2018)	Egypt	40	20	11.6 (3.1)	N.a.	NT	Yes	Internalizing (CBCL) Anxious/Depressed (CBCL) Attention problems (CBCL) Social problems (CBCL)	57.5 (8.5) 55.6 (10.9) 57.4 (7.2) 60.3 (7.1)	51.7 (2.3) 45.8 (2.6) 52.9 (3.2) 54.1 (4.5)	8
Al‐Zaben and Sehlo (2014)	Saudi Arabia	65	40	9.3 (1.8)	24.8	NT	Yes	Depression (CDI)	13.7 (1.7)	8.0 (1.3)	7
Kanata et al. (2016)	Japan	407	3997	10.2 (0.3)	47.1	NT	No	Emotional symptoms (SDQ) Hyperactivity‐inattention (SDQ) Conduct problems (SDQ) Peer relationship problems (SDQ)	1.8 (1.9) 3.8 (2.3) 2.2 (1.7) 1.8 (1.7)	1.6 (1.7) 3.0 (2.1) 1.8 (1.5) 1.5 (1.6)	8
Ucer and GumuA (2013)	Turkey	101	38	11.0 (2.0)	37.3	NT	No	Sleep (PSQI) Depression (CDI)	2.6 (2.5) 11.7 (6.1)	1.2 (1.1) 7.0 (3.4)	6
Akyüz et al. (2016)	Turkey	38	46	10.8 (7.1)	52.0	NT	No	Withdrawn behavior (CBCL) Somatic complaint (CBCL) Anxious/Depressed (CBCL) Social problems (CBCL) Thought problems (CBCL) Attention problems (CBCL) Delinquent behavior (CBCL) Aggressive behavior (CBCL)	3.2 (2.8) 0.8 (1.1) 6.3 (4.4) 2.8 (2.5) 1.1 (1.4) 5.6 (3.3) 2.1 (2.8) 8.9 (7.6)	2.9 (2.5) 0.7 (0.9) 5.5 (3.8) 1.5 (1.7) 1.0 (1.3) 4.0 (2.8) 1.7 (2.1) 7.4 (6.0)	5
Wagner et al. (2015)	Germany	9	44	8.3 (2.0)	43.7	NT	No	Internalizing (CBCL) Externalizing (CBCL)	59.3 (10.1) 54.1 (7.4)	50.3 (9.5) 49.7 (9.7)	7
Koca et al. (2014)	Turkey	38	46	10.8 (3.5)	52.0	NT	N.a.	Self‐esteem (PHCSCS) Depression (CDI)	54.3 (13.0) 10.4 (4.3)	63.4 (9.1) 7.1 (4.4)	6
Erdogan et al. (2008)	Turkey	53	303	6.0 (1.0)	51.0	N.a.	N.a.	Internalizing (CBCL) Externalizing (CBCL) Withdrawn behavior (CBCL) Somatic complaint (CBCL) Anxious/Depressed (CBCL) Social problems (CBCL) Thought problems (CBCL) Attention problems (CBCL) Delinquent behavior (CBCL) Aggressive behavior (CBCL)	56.3 (10.2) 53.0 (8.6) 54.9 (6.2) 54.6 (8.0) 58.3 (7.7) 55.0 (5.4) 57.6 (6.4) 57.8 (6.4) 54.3 (7.7) 55.3 (7.5)	54.4 (10.1) 50.1 (10.2) 54.8 (6.5) 54.8 (6.9) 57.3 (7.0) 5.4 (7.6) 7.3 (24.2) 7.3 (12.6) 6.6 (17.0) 8.0 (11.7)	6
van Hoecke et al. (2004)	Belgium	84	70	10.1 (2.0)	36.5	CB	N.a.	Withdrawn behavior (CBCL) Somatic complaint (CBCL) Anxious/Depressed (CBCL) Social problems (CBCL) Anxiety (SAS‐C) Anxiety (STAI‐C) Depression (SDQ‐C) Self‐esteem (SPPC)	2.8 (2.7) 1.7 (2.3) 5.7 (5.2) 3.0 (2.9) 10.5 (8.3) 33.3 (5.8) 1.8 (1.9) 18.3 (4.0)	1.8 (2.3) 1.3 (1.9) 3.8 (4.3) 2.2 (2.6) 11.0 (10.5) 33.1 (8.2) 2.3 (2.7) 18.9 (4.0)	7
Kanaheswari et al. (2012)	Malaysia	63	63	10.3 (6.0)	47.6	NT	N.a.	Self‐esteem (ITIA)	28.3 (16.9)	35.7 (16.8)	8
Natale et al. (2009)	Germany	49	32	7.0 (5.0)	42.9	CB	N.a.	Self‐esteem (PHCSCS)	49.3 (7.2)	51.5 (7.3)	7
Ma et al. (2020)	China	158	168	N.a.	48.8	NT	No	Self‐esteem (PHCSCS)	55.2 (6.1)	56.6 (5.2)	7
Birdal and Doğangün (2016)	Turkey	30	30	9.21 (N.a.)	N.a.	CB	No	Anxious/Depressed (CBCL) Somatic complaint (CBCL) Social problems (CBCL) Thought problems (CBCL) Aggressive behavior (CBCL)	4.2 (3.3) 5.7 (4.6) 4.0 (2.8) 2.1 (2.5) 3.6 (4.2)	1.6 (1.8) 2.3 (2.3) 1.9 (1.8) 0.7 (1.1) 0.8 (1.7)	5
Desta et al. (2007)	Ethiopia	511	1890	10.1 (N.a.)	51.2	N.a.	No	Disruptive behavior (DICA) Anxiety (DICA)	2.7% 11.9%	1.1% 7.6%	6
Van Hoecke et al. (2006)	Belgium	141	155	8.5 (1.8)	38.5	CB	No	Internalizing (CBCL) Externalizing (CBCL) Attention problems (DBDRS) Hyperactive problems (DBDRS)	57.4 (10.8) 50.3 (11.1) 7.5 (5.8) 7.2 (6.4)	52.5 (10.7) 49.2 (10.0) 5.7 (5.4) 4.7 (5.4)	6
Ş et al. (2019)	Turkey	56	42	14.0 (1.7)	22.9	NT	No	Anxiety (SCARED) Depression (CDI) Social anxiety (SAS‐A)	31.7 (16.1) 14.7 (7.7) 48.7 (15.0)	26.3 (13.3) 16.1 (8.6) 35.8 (11.6)	7
Equit et al. (2013)	Germany	279	1799	6.2 (0.4)	48.8	NT	No	Anxious/Depressed (CBCL)	57.5 (8.1)	56.0 (7.2)	7
Ertan et al. (2009)	Turkey	44	27	10.8 (3.1)	67.6	NT	No	Sleep (PSQI)	3.6 (2.1)	3.4 (1.7)	6
Hamed et al. (2021)	Egypt	80	60	13.0 (1.3)	37.9	NT	No	Emotional symptoms (SDQ) Hyperactivity‐inattention (SDQ) Conduct problems (SDQ) Peer relationship problems (SDQ)	7.2 (1.6) 6.9 (1.8) 8.6 (1.7) 7.6 (1.7)	3.8 (0.8) 3.0 (0.8) 4.5 (1.0) 3.2 (1.0)	8

Abbreviations: CB, Combined; CBCL, Child Behavior Checklist; CDI, Children's Depression Inventory; DBDRS, Disruptive Behavior Disorder Rating Scale; DYPSIS, Diagnostic Interview for Mental Disorders in Children and Adolescent; EN, Enuresis; HC, Healthy Controls; ITIA, I Think I Am Test; DICA, Diagnostic Interview for Children and Adolescents; MASC, Multidimensional Anxiety Scale for Children; MSCS, Multidimensional Self Concept Scale; NOS, Newcastle Ottawa Score; NT, Nocturnal; PFC, Preschool Feelings Checklist; PHCSCS, Piers‐Harris Children's Self‐Concept Scale; PSQI, Pittsburgh Sleep Quality Index; SAS‐A, Social Anxiety Scale for Adolescents; SAS‐C, Social Anxiety Scale for Children; SCARED, Screen for Child Anxiety Related Disorders; SD, Standard Deviation; SDQ, Strengths and Difficulties Questionnaire; SPPC, Self‐Perception Profile for Children; STAI‐C, State Trait Anxiety Inventory for Children.

**TABLE 2:** Articles included in the systematic review for the encopresis sample.

Author year	Country	N EC	N HC	Age mean (SD)	% Female	Domain (scale)	EC mean (SD)/cutoff %	HC mean (SD)/cutoff %	NOS
Niemczyk et al. (2014)	Germany	12	1466	5.7 (0.4)	50.2	Externalizing disorders (DYSIPS)	50%	9.5%	6
Mattheus et al. (2021)	Germany	28	43	5.42 (0.8)	45.7	Internalizing (CBCL) Externalizing (CBCL) Depression (PFC)	52.33 (9.07) 52.56 (11.7) 1.18 (1.58)	44.76 (10.51) 44.33 (11.7) 0.7 (1.29)	7
Akça et al. (2011)	Turkey	30	30	N.a.	43.7	Depression (K‐SADS) Anxiety (K‐SADS) ADHD (K‐SADS) OD disorder (K‐SADS)	23.3% 40.0% 36.7% 40.0%	6.6% 33.3% 16.7% 3.33%	5
Wagner et al. (2015)	Germany	17	44	8.25 (2.0)	30.1	Internalizing (CBCL) Externalizing (CBCL)	61.44 (11.1) 64.56 (12.5)	50.27 (9.5) 49.73 (9.7)	7
Cox et al. (2002)	USA	86	62	9.16 (2.23)	30.1	Attention problems (CBCL) Social problems (CBCL) Withdrawn behavior (CBCL) Delinquent behavior (CBCL) Aggressive behavior (CBCL) Self‐esteem (PHCSCS)	58.88 (8.29) 87.8 (8.71) 57.0 (8.47) 57.0 (7.21) 57.45 (8.24) 61.29 (12.61)	23.62 (5.79) 53.57 (6.0) 52.11 (3.7) 52.36 (4.91) 54.39 (7.46) 63.43 (10.03)	6
Landman et al. (1986)	USA	31	35	11.0 (0.9)	37.5	Self‐esteem (PHCSCS)	18.5 (3.8)	20.7 (4.3)	5
Owens‐stively (1987)	USA	10	10	9.0 (1.8)	30.0	Self‐esteem (PHCSCS)	59.4 (n.a.)	69.5 (n.a.)	4

Abbreviations: CBCL, Child Behavior Checklist; DYPSIS, Diagnostic Interview for Mental Disorders in Children and Adolescent; EC, Encopresis; HC, Healthy Controls; K‐SADS, Kiddie Schedule for Affective Disorders and Schizophrenia; NOS, Newcastle Ottawa Score; PFC, Preschool Feelings Checklist; PHCSCS, Piers‐Harris Children's Self‐Concept Scale; SD, Standard Deviation.

Second, we performed meta‐analyses where data allowed for it. The comparison of effect sizes in each group was calculated using effect size (ES) formula. A negative ES indicates the elimination disorders group has more internalizing and/or externalizing problems than the control, except for the Piers‐Harris Children's Self‐Concept Scale, where a positive ES indicates the elimination disorders group has lower self‐concept. Effect sizes were calculated using the means, standard deviations (SDs), and sample size for the outcomes of interest for each sample. Meta‐regressions were performed to determine the effect of the (a) age, (b) sex, and (c) NOS score on the outcomes of interest. Heterogeneity among studies was assessed using the Q statistic, with the proportion of the total variability in effect size estimates evaluated using the *I* ^2^ index, classifying the heterogeneity as low (*I* ^2^ = 25%), medium (*I* ^2^ = 50%), and high (*I* ^2^ = 75%) (Lipsey & Wilson, 2009). The random‐effects model was used. Publication bias was assessed by visually inspecting funnel plots, and the trim and fill method (Duval & Tweedie, 2000) was used when funnel plot's asymmetry suggested a significant publication bias.

All analyses were conducted within R 1.4.1106 (R Foundation for Statistical Computing, 2021). The significance level was set at a *p* < 0.05, two‐sided.

## RESULTS

The literature search yielded 3922 citations through electronic database, which were screened for eligibility; 121 articles were assessed in full text, and 85 were excluded. The final database for the systematic review and meta‐analysis included 36 studies, on which 32 report results for enuretic children and 7 for encopretic children, as it can be seen in Figure S1 (PRISMA Flow Diagram). Studies included samples from 13 countries in four continents: Europe (36.1%), Asia (47.2%, being 70.6% of them from Turkey), Africa (8.3%), and North America (8.3%). Mean NOS score for the included studies was 6.4 ± 1.1. 16.6% and 44.4% of the included studies presented a high or a moderate certainty of evidence, respectively, while 38.9% of them showed either a low or very low certainty of evidence (See Table S4). *Enuresis*.

Data were extracted from 32 studies for a total sample size of 3244 patients (mean age = 9.4; 43.84% female) and 11,277 controls (mean age = 10.1; 48.98% female). 62.5% of the studies reported on children with nocturnal enuresis, while 28.1% also included samples with combined enuresis (nocturnal and diurnal). 15.6% of the studies included children already receiving medical treatment for their elimination disorder. The included studies reported on a wide range of both internalizing and externalizing behavior (Table 1).

### Internalizing problems meta‐analyses

Enough data was found to perform a meta‐analysis on 8 scales or subscales reporting on internalizing problems: Piers‐Harris Self‐Concept Scale (Piers, 1984) (a validated scale used to measure self‐concept), Children's Depression Inventory (Kovacs, 1981) (measuring depressive symptoms), Pittsburgh Sleep Quality Index (Buysse et al., 1989) (measuring sleep problems), Child Behavior Checklist (Achenbach & Edelbrock, 1983) (CBCL) Internalizing broad band factor, and four other CBCL subscales: Anxiety/Depression, Somatic complaints, Withdrawn behavior and Thought problems.

**FIGURE 1:**
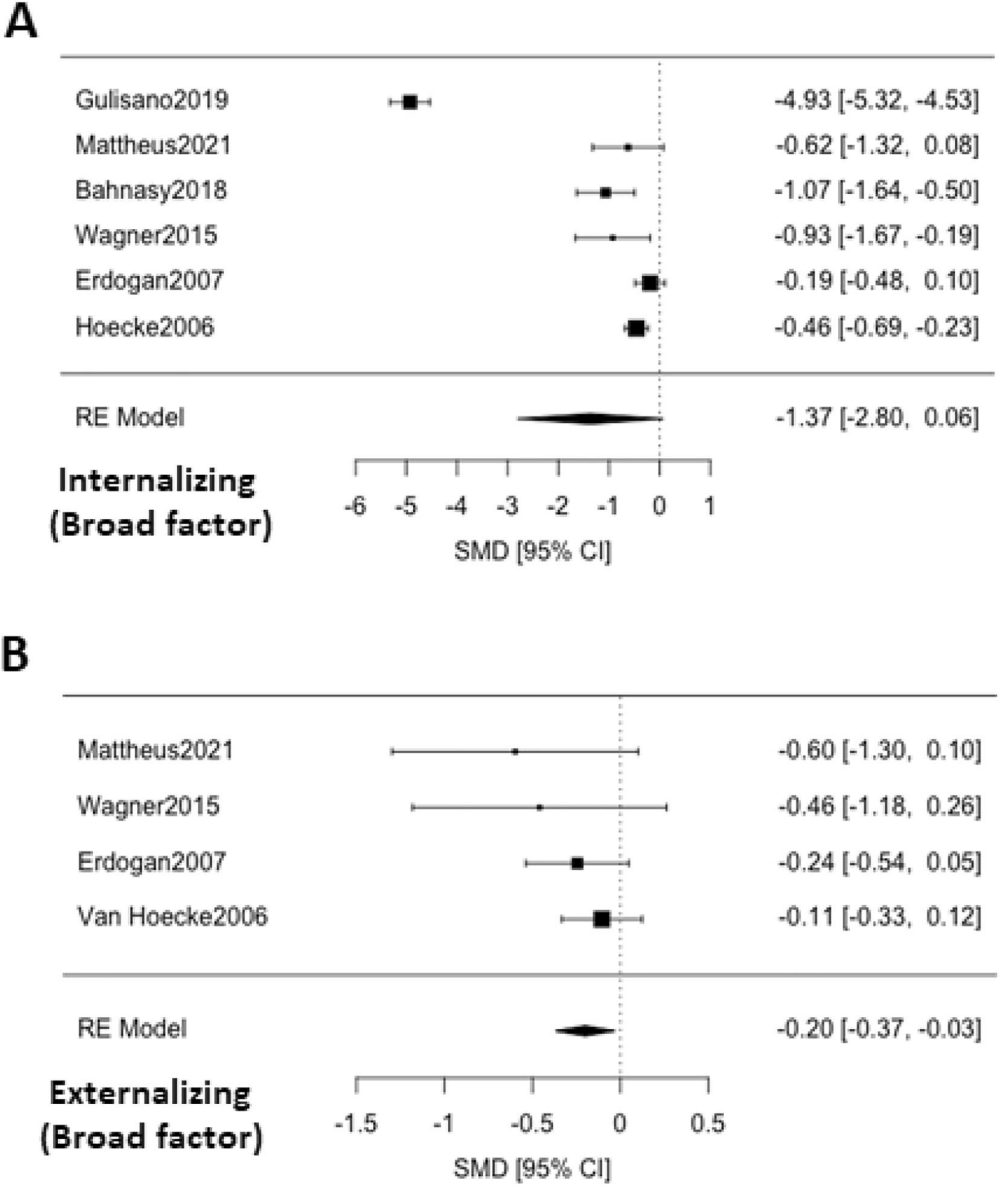
Forest plots for the standardized mean differences (SMD) of CBCL broad‐band internalizing scale (A) and CBCL broad‐band externalizing scale (B) in children with enuresis versus healthy controls.

As it can be seen in Figure 1A and Table 3A, internalizing behaviors measured with CBCL's broad‐band factor did not significantly differ between children with enuresis and their non‐enuretic peers (ES = −1.37; 95% CI ‐2.80; 0.06; *p* = 0.061). As for specific internalizing behaviors, only two of the meta‐analyzed problems significantly differed between groups. Self‐concept was significantly lower in children with enuresis (ES = 0.42; 95% CI 0.08; 0.76; *p* = 0.017), while CBCL Thought Problems subscale scored higher in the enuretic group (ES = −0.26; 95% CI ‐0.43; −0.09; *p* = 0.003). Both groups did not significantly differ in Withdrawn behavior, Somatic complaints or Anxiety/Depression subscales, nor for Depression or Sleep problems (See Figure S2).

**TABLE 3:** Results summary for internalizing (A) and externalizing (B) problems in children with enuresis versus healthy controls.

A – INTERNALIZING PROBLEMS										
								Meta‐regressions		
Domain	Scale	k (N)	SMD [95% CI] (*p* value)	Q (*p* value)	I^2^ (%)	Publication bias	Corrected SMD [95% IC] (*p* value)	Age (SE) (*p* value)	NOS (SE) (*p* value)	Sex (SE) (*p* value)
Internalizing symptoms	CBCL	6 (1217)	−1.37 [−2.80; 0.06] (*p* 0.061)	431.92 (*p* 0.0001)	98.69	Yes	−1.90 [−3.14; −0.65] (*p* 0.0028)	−0.03 (0.20) (*p* 0.87)	−4.15 (0.90) (*p* 0.0001)*	0.02 (0.05) (*p* 0.67)
Depression	CDI	6 (1055)	−0.98 [−2.02; 0.07] (*p* 0.068)	110.88 (*p* 0.0001)	98.27	No	‐	0.50 (0.23) (*p* 0.03)*	−1.21 (0.55) (*p* 0.03)*	0.03 (0.04) (*p* 0.47)
Anxiety/Depression	CBCL	7 (1253)	0.68 [−1.35; 2.72] (*p* 0.510)	479.82 (*p* 0.0001)	99.59	No	‐	−0.01 (0.01) (*p* 0.48)	−2.02 (6.08) (*p* 0.74)	1.0 (0.00) (*p* 0.99)
Somatic complaints	CBCL	6 (1193)	−0.54 [−1.25; 0.16] (*p* 0.132)	212.03 (*p* 0.0001)	96.82	Yes	−0.67 [−1.32; −0.02] (*p* 0.042)	−0.38 (0.17) (*p* 0.02)*	−1.08 (0.52) (*p* 0.04)*	−0.02 (0.05) (*p* 0.67)
Self‐concept	PHCSCS	3 (491)	0.42 [0.08; 0.76] (*p* 0.017)*	5.27 (*p* 0.07)	62.65	No	‐	N.a.	N.a.	N.a.
Sleep	PSQI	3 (261)	−0.77 [−1.61; 0.08] (*p* 0.075)	13.67 (*p* 0.001)	88.59	No	‐	N.a.	N.a.	N.a.
Withdrawn behavior	CBCL	5 (1039)	−1.30 [−3.17; 0.56] (*p* 0.171)	461.83 (*p* 0.0001)	99.32	No	‐	N.a.	N.a.	N.a.
Thought problems	CBCL	5 (1039)	−0.26 [−0.43; −0.09] (*p* 0.003)*	7.26 (*p* 0.12)	38.24	No	‐	N.a	N.a.	N.a.

B – EXTERNALIZING PROBLEMS.										
								Meta‐regressions		
Domain	Scale	k (N)	SMD [95% CI] (*p* value)	Q (*p* value)	I^2^ (%)	Publication bias	Corrected SMD [95% IC] (*p* value)	Age (SE) (*p* value)	NOS (SE) (*p* value)	Sex (SE) (*p* value)
Externalizing symptoms	CBCL	4 (757)	−0.20 [−0.37; −0.03] (*p* 0.020)*	2.49 (*p* 0.48)	0.00	Yes	−0.16 [−0,31; 0.002] (*p* 0.054)	0.12 (0.15) (*p* 0.41)	N.a.	0.02 (0.03) (*p* 0.65)
Attention problems	CBCL	5 (1039)	−2.48 [−6.24; 1.28] (*p* 0.196)	639.93 (*p* 0.0001)	99.80	No	‐	N.a.	N.a.	N.a.
Attention	DBDRS	3 (791)	−0.37 [−0.51; −0.22] (*p* 0.0001)*	2.08 (*p* 0.35)	0.00	No	‐	N.a.	N.a.	N.a.
Aggressive behavior	CBCL	5 (1039)	−0.33 [−0.62; −0.04] (*p* 0.025)*	19.97 (*p* 0.0005)	78.41	Yes	−0.24 [−0,55; 0,07] (*p* 0.127)	N.a.	N.a.	N.a.
Social problems	CBCL	7 (1253)	−0.39 [−0.58; −0.21] (*p* 0.0001)*	13.17 (*p* 0.04)	57.07	Yes	−0.35 [−0.58; −0.12] (*p* 0.002)	−0.04 (0.06) (*p* 0.48)	0.17 (0.20) (*p* 0.38)	−0.01 (0.02) (*p* 0.76)
Delinquent behavior	CBCL	4 (979)	−0.07 [−0.33; 0.20] (*p* 0.626)	14.54 (*p* 0.002)	74.88	Yes	0.1047 [−0.18; 0.38] (*p* 0.464)	N.a.	N.a.	N.a.

*Note*: Where publication bias exists, a Corrected SMD is proposed using the trim and fill method.

Abbreviations: CBCL, Child Behavior Checklist; CDI, Children's Depression Inventory; DBDRS, Disruptive Behavior Disorder Rating Scale; IC, Confidence Interval; PHCSCS, Piers‐Harris Children's Self‐Concept Scale; PSQI, Pittsburgh Sleep Quality Index; SE, Standard Error; SMD, Standardized Mean Difference.

**FIGURE 2:**
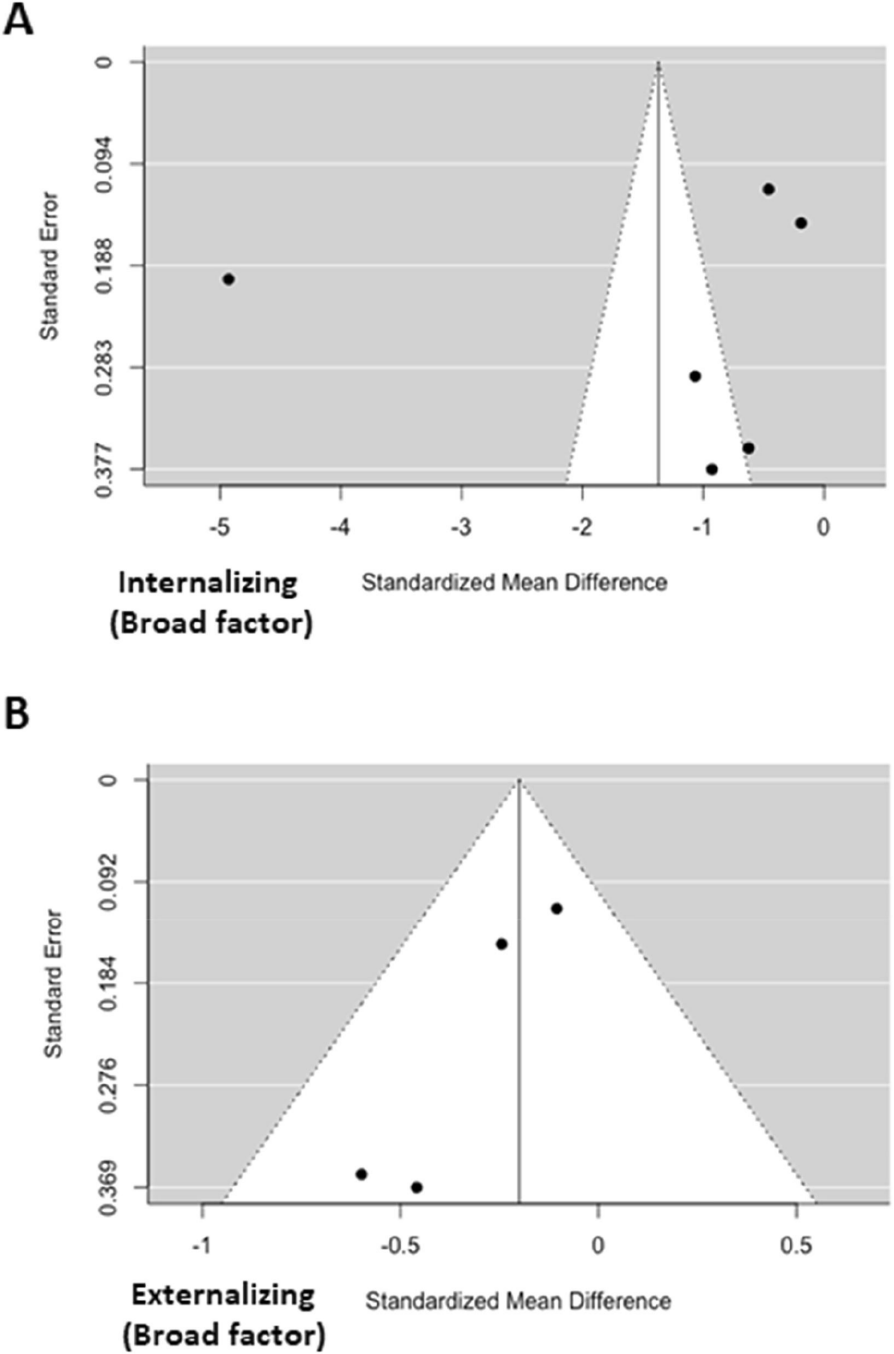
Funnel plots for the CBCL broad‐band internalizing scale meta‐analysis (A) and CBCL broad‐band externalizing scale meta‐analysis (B).

Heterogeneity was significant across all of the meta‐analyzed subscales, except for Self‐concept and Thought problems. Funnel plots suggested the presence of a publication bias for Internalizing symptoms broad band factor (corrected ES = −1.90; 95% CI ‐3.14; −0.65; *p* = 0.003) and Somatic complaints subscales (corrected ES = −0.67; 95% CI ‐1.32; −0.02; *p* = 0.042) (See Figure S3) (Figure 2). After performing trim and fill method corrections, both subscales significantly differed between groups.

There was not enough data available in the original studies to perform meta‐regressions on those subscales where the two groups significantly differed.

### Externalizing problems meta‐analyses

Enough data was found to perform a meta‐analysis on 6 subscales reporting on internalizing problems: Disruptive Behavior Disorder Rating Scale's (DBDRS) Attention subscale (Greenslade et al., 1992) (measuring attention problems), CBCL Externalizing symptoms broad band factor, and four other CBCL subscales: Attention problems, Aggressive behavior, Social problems and Delinquent behavior.

As shown in Figure 1B and Table 3B, externalizing behaviors measured with CBCL's broad‐band factor scored significantly higher in children with enuresis compared with healthy controls (ES = −0.20; 95% CI ‐0.37; −0.03; *p* = 0.020). Three other externalizing problems subscales also significantly differed between groups, in all cases presenting higher problematic scores for the enuretic group: Attention (ES = −0.37; 95% CI ‐0.51; −0.22; *p* = 0.0001); Aggressive behavior (ES = −0.33; 95% CI ‐0.62; −0.04; *p* = 0.025); Social problems (ES = −0.39; 95% CI ‐0.58; −0.21; *p* = 0.0001). That was not the case for Delinquent behavior and Attention problems measured by CBCL (Figure S4).

Again, heterogeneity was significant across all of the meta‐analyzed subscales except for the Externalizing broad band factor and Attention (measured by DBDRS). Funnel plots suggested the presence of a publication bias for 4 subscales: Externalizing symptoms broad band factor (corrected ES = −0.16; 95% CI ‐0.31; 0.0002; *p* = 0.054), Aggressive behavior (corrected ES = −0.24; 95% CI ‐0.55; 0.07; *p* = 0.127), Social problems (corrected ES = −0.35; 95% CI ‐0.58; −0.12; *p* = 0.002) and Delinquent behavior (corrected ES = 0.105; 95% CI ‐0.18; 0.38; *p* = 0.464) (See Figure S5).

Meta‐regressions were performed where available data allowed for it: age, NOS score and sex were not significantly associated with Externalizing symptoms nor Delinquent behavior.

### Encopresis

After allowing for overlapping studies, data were extracted from 7 studies for a total sample size of 214 patients (mean age = 8.6; 36.24% female) and 1689 controls (mean age = 8.6; 47.82% female). The included studies reported on a range of both externalizing and internalizing behavior, as it can be seen in Table 2. No domain provided enough data to be meta‐analyzed. However, all the analyzed domains reflected more internalizing and externalizing problems in the encopretic group compared with their healthy peers.

## DISCUSSION

The results of this systematic review and meta‐analysis reveal important findings. We have found evidence that there is a significant increase in some internalizing and externalizing symptoms and behaviors in those children with elimination disorders. However, original data available to meta‐analyze was limited and presented significant heterogeneity, as well as the existence of a publication bias for most of the studied domains.

The studies included reported on children from 13 countries across four continents. Surprisingly, there were no articles reporting on enuretic children from North America, while an overrepresentation of non‐overlapping Turkish samples (12 articles in total) was found. Nocturnal enuresis prevalence seems to vary significantly across countries, ranging from around 20% in Turkey (Gunes et al., 2009) to 4.5% in the United States (Shreeram S et al., 2009), which, along with cultural differences, could explain this atypical geographic distribution of the available literature.

A lower self‐concept was found in those children with enuresis compared with their non‐enuretic peers. This finding is consistent with previous reports in the literature, although some controversy exists. Most of the articles included in the systematic review reported lower self‐esteem scores for the elimination disorders group, as do several other studies that did not meet the criteria to be included. This lower self‐concept seems to be worsened by a growing number of failed treatments (Theunis et al., 2002), and significantly improves after enuresis is effectively treated, reaching the same levels as the HC group (Hägglöf et al., 1997). However, Natale did not report significant differences among groups, although it was a small clinical sample of exclusively nocturnal enuresis patients (Natale et al., 2009). A previous review on this topic including four small studies also found no evidence indicating that nocturnal enuresis led to a low self‐esteem. However, its effective treatment again seemed to result in a self‐concept improvement (Redsell & Collier, 2001).

As for other internalizing symptoms, we found no evidence that depression or anxiety scores might be increased in children with an elimination disorder. This differs substantially from previous reports in literature. Although some controversy exists regarding the presence of anxiety in enuretic children (Gulisano et al., 2016) (with some authors even finding lower anxiety rates compared to HC subjects (Koca et al., 2014)), literature is almost unanimous in pointing higher depressive symptoms in enuretic children (Gulisano et al., 2016; Koca et al., 2014; Ş et al., 2019; Ucer & GumuA, 2013), and some authors associated it with punishment for bedwetting (Al‐Zaben & Sehlo, 2014). However, some of the included samples are from clinical settings, possibly including especially symptomatic children and thus, incurring in a selection bias.

Regarding externalizing symptoms, our findings show increased symptomatic scores for almost all the studied domains, including attention problems, aggressive behavior and social problems. This is in line with previous evidence (Wagner et al., 2015). However, it is not that clear those increased scores translate to clinically relevant behavioral problems (Erdogan et al., 2008). Moreover, many authors reported these behavioral differences disappeared once some variables such as socioeconomic status (SES), male gender or school performance were added to the analyses (Ozden et al., 2007; VAN HOECKE et al., 2003). Biological substrate also seems to play an important role in behavioral alterations; in fact, some authors have recently found abnormal neural responses to emotional stimuli, increased excitability and reduced inhibitory processing in individuals with nocturnal enuresis history (Khedr et al., 2015; Wang et al., 2015), similar to those findings reported in neuropsychiatric conditions such as ADHD or Tourette syndrome (Ma et al., 2016; Morand‐Beaulieu et al., 2017).

Far less information has been found on encopresis and its psychological and behavioral correlates. The 7 articles included in our systematic review show a lower self‐esteem in patients with encopresis, as well as higher symptomatic scores for both internalizing and externalizing problems. Previous literature shows consistent findings, and often notes poorer family functioning in families where children with encopresis are raised (Akça et al., 2011; Lisboa et al., 2008).

Last but not least, it is necessary to point out that a significant proportion of the studies included in the analysis appear to be lacking in terms of their quality. 55% of the included studies presented a NOS score of 6 or below, which suggests there may be important limitations in the study design or methodology and, consequently, a higher risk of bias, thus compromising the reliability and validity of its findings. Along with this issues, great heterogeneity was found across most of the studied domains. Also, several of the internalizing symptoms studied (such as the CBCL Internalizing broad band factor or the Somatic complaints subscale) presented significant differences among groups once the trim and fill method was applied where funnel plots suggested a publication bias. In the case of some externalizing behaviors (as the CBCL Externalizing broad band factor, among others) the opposite occurred, with these differences disappearing when the results were corrected by the aforementioned method. This speaks of the existence of highly relevant biases in the available literature. Many studies included small samples, most of them from clinical settings, therefore possibly subjected to selection bias. Some authors also used siblings of enuretic children, or children with other chronic disorders, as healthy controls, thus potentially incurring in other, important biases. All of the above suggests the need to perform further, unbiased research on the topic.

Shaffer described four possible ways to interpret associations between enuresis and psychological symptoms, also applicable for encopresis (Shaffer, 1973). First, there is no causal relationship between the two conceptions because their association is the result of chance. Second, psychological elements like temperament or unfavorable life events cause enuresis. Third, psychological symptoms represent a consequence of enuresis. And fourth, both psychological problems and enuresis are related to common risk factors, such as the aforementioned low socio‐economic status or male sex. These four explanations are not mutually exclusive, and often complement each other. Although further longitudinal research is needed to explore the causal nature of these two entities, it is clear emotional and behavioral symptoms are to be considered in the evaluation and treatment of any children with an elimination disorder.

### Strengths and limitations

To the best of the authors' knowledge, this is the first systematic review and meta‐analysis to date to assess the relationship between elimination disorders and internalizing and externalizing problems. It evaluates a wide range of behaviors present in children with encopresis and enuresis, all of them measured through questionnaires with high validity and reliability.

Notwithstanding all the above, some important limitations must be mentioned.

Apart from the intrinsic limitations of the available literature (as detailed above), there was a significant lack of information on the patients studied, and therefore not enough data was available to perform meta‐regressions on several of the analyzed domains. Where analyses were possible, they failed to provide an explanation for the great heterogeneity found in the analyses. This could mean the results of the meta‐analysis could be related to, and partially explained by, third variables not studied, such as the type of enuresis (primary or secondary, diurnal or combined), the presence or absence of secondary physical punishment (Al‐Zaben & Sehlo, 2014), or socioeconomic status (VAN HOECKE et al., 2003). Likewise, elimination disorders, as well as their management by the child's caregivers, are strongly influenced by culture (Hurl, 2011). All of them are factors that have been extensively related in the past to both internalizing and externalizing symptoms as well as to elimination disorders, but it has not been possible to evaluate these potential moderators due to the lack of data in the original studies. Further research should consider and assess these issues, ideally using large, representative samples of children with elimination disorders and their healthy peers.

### Implications for research and clinical practice

This systematic review and meta‐analysis holds several implications regarding future research and clinical practice. Practitioners should be aware that children with an elimination disorder may have internalizing and externalizing problems, as well as impaired self‐concept. This entails the need to consider these parameters when evaluating a child with encopresis and/or enuresis. Likewise, these findings seem to indicate that these disorders should not be approached from a purely behavioral or pharmacological point, but that psychological support and socio‐emotional training should be offered to both affected children and their main caregivers, in order to reduce and prevent the appearance of these symptoms.

Also, as discussed above, further research is needed to clarify for which particular internalizing and externalizing symptoms enuretic and encopretic children are at risk, as well as which factors might be moderating them. In order the study such symptoms, the authors suggest the need to use standardized definitions of elimination disorders, ideally differentiating between its subtypes: primary or secondary, diurnal or combined, as they could each have different psychopathological implications. Also, the authors consider CBCL to be the most comprehensive questionnaire to assess internalizing and externalizing symptoms in children, according to its validity and focus in symptomatologic domains.

## CONCLUSION

Children with an elimination disorder presented higher scores for externalizing symptoms (including attention alterations, aggressive behavior and social problems), as well as a significantly lower self‐concept. On the other hand, no evidence has been found that depression or anxiety scores might be increased in children with an elimination disorder. Further, unbiased research is needed to clarify which factors might be moderating these findings.

## AUTHOR CONTRIBUTIONS

**Claudia Aymerich**: Conceptualization; Data curation; Investigation; Methodology; Resources; Validation; Visualization; Writing – original draft. **Borja Pedruzo**: Conceptualization; Writing – original draft. **Malein Pacho**: Conceptualization; Data curation; Methodology. **Jon Herrero**: Data curation. **Maria Laborda**: Data curation. **Gonzalo Salazar De Pablo**: Conceptualization; Writing – review & editing. **Eva Sesma**: Writing – review & editing. **Aranzazu Fernández‐Rivas**: Writing – review & editing. **Ana Catalan**: Conceptualization; Investigation; Software; Supervision; Writing – review & editing. **Miguel Ángel González‐Torres**: Supervision; Writing – review & editing.

## CONFLICT OF INTEREST STATEMENT

The authors have declared that they have no competing or potential conflicts of interest.

## ETHICAL CONSIDERATIONS

No ethical approval was required for this research review.

## Supporting information

Supporting Information S1

## Data Availability

The data that support the findings of this study are available from the corresponding author upon reasonable request.
